# Insulin-stimulated glucose uptake in skeletal muscle, adipose tissue and liver: a positron emission tomography study

**DOI:** 10.1530/EJE-17-0882

**Published:** 2018-03-07

**Authors:** Miikka-Juhani Honka, Aino Latva-Rasku, Marco Bucci, Kirsi A Virtanen, Jarna C Hannukainen, Kari K Kalliokoski, Pirjo Nuutila

**Affiliations:** 1 Turku PET Centre, University of TurkuTurku, Finland; 2 Turku PET Centre, Åbo Akademi UniversityTurku, Finland; 3 Turku PET Centre, Institute of Public Health and Clinical NutritionUniversity of Eastern Finland, Kuopio, Finland; 4 Department of EndocrinologyTurku University Hospital, Turku, Finland

## Abstract

**Objective:**

Insulin resistance is reflected by the rates of reduced glucose uptake (GU) into the key insulin-sensitive tissues, skeletal muscle, liver and adipose tissue. It is unclear whether insulin resistance occurs simultaneously in all these tissues or whether insulin resistance is tissue specific.

**Design and methods:**

We measured GU in skeletal muscle, adipose tissue and liver and endogenous glucose production (EGP), in a single session using ^18^F-fluorodeoxyglucose with positron emission tomography (PET) and euglycemic–hyperinsulinemic clamp. The study population consisted of 326 subjects without diabetes from the CMgene study cohort.

**Results:**

Skeletal muscle GU less than 33 µmol/kg tissue/min and subcutaneous adipose tissue GU less than 11.5 µmol/kg tissue/min characterized insulin-resistant individuals. Men had considerably worse insulin suppression of EGP compared to women. By using principal component analysis (PCA), BMI inversely and skeletal muscle, adipose tissue and liver GU positively loaded on same principal component explaining one-third of the variation in these measures. The results were largely similar when liver GU was replaced by EGP in PCA. Liver GU and EGP were positively associated with aging.

**Conclusions:**

We have provided threshold values, which can be used to identify tissue-specific insulin resistance. In addition, we found that insulin resistance measured by GU was only partially similar across all insulin-sensitive tissues studied, skeletal muscle, adipose tissue and liver and was affected by obesity, aging and gender.

## Introduction

Insulin resistance is a major feature in the development and pathophysiology of type 2 diabetes. In healthy individuals, skeletal muscle takes the major part of glucose uptake (GU) during hyperinsulinemia (about 75–80%), whereas the proportion of GU into adipose tissue and liver is substantially smaller (1). Individuals with normal weight and normal glucose tolerance are highly sensitive to insulin in skeletal muscle, adipose tissue and liver (2, 3, 4), whereas obese individuals and individuals with type 2 diabetes are insulin resistant (4, 5, 6). Decreased muscle GU, increased endogenic hepatic glucose production (EGP) and impaired insulin secretion contribute to hyperglycemia and type 2 diabetes (1).

Activation of the phosphoinositol 3-kinase/AKT2 pathway results in translocation of insulin-sensitive glucose transporter GLUT4 (SLC2A4) to the plasma membrane and in an increase of glucose transport into muscle and adipose tissue (7, 8). In the liver, activated AKT inhibits the rate-controlling enzymes of gluconeogenesis resulting in the suppression of EGP and promotes glycogen synthesis (9, 10). Additionally, increased lipolysis from white adipose tissue promotes hepatic insulin resistance by increasing hepatic gluconeogenesis via an increased free fatty acid (FFA) flux into the liver (11). Glycogen is the main repository for GU in the liver (12). Additionally, FFAs, inflammatory cytokines and organokines can affect insulin sensitivity in various tissues (13, 14, 15).

Insulin sensitivity in humans has been previously addressed by evaluating whole body GU, EGP or suppression of lipolysis (16, 17, 18, 19, 20). The use of positron emission tomography (PET) with a positron-emitting glucose analog, ^18^F-fluorodeoxyglucose ([^18^F]FDG), allows the direct measurement of the rates of GU in multiple insulin-sensitive tissues. Combining this method with the measurements of whole body insulin sensitivity, the euglycemic–hyperinsulinemic clamp (21), allows us to directly assess tissue-specific GU or insulin sensitivity. Even though muscle GU is responsible for most of the whole body GU during euglycemic–hyperinsulinemic clamp, contribution of liver is higher after meal when liver takes up approximately one-third of an ingested glucose load and EGP is suppressed (12). Furthermore, contribution of adipose tissue on whole body GU is larger when fat mass is high (22, 23). Therefore, it is important to understand whether insulin resistance to GU and EGP suppression occur simultaneously in all these tissues or whether insulin resistance is tissue specific. To address this question, we collected a large cohort of non-diabetic individuals whose skeletal muscle, liver and adipose tissue insulin sensitivity have been measured using PET, ^18^F-FDG and the euglycemic–hyperinsulinemic clamp.

## Subjects and methods

### Subjects

The subjects were 326 volunteers who had previously participated in PET studies at the Turku PET Centre (Turku, Finland) and who did not have diabetes according to the American Diabetes Association criteria (24) (Table 1). Women were older than men (*P* < 0.001) but no difference was observed in body mass index (BMI). All subjects gave a written informed consent. The study protocol was approved by the Ethics Committee of the Hospital District of South-West Finland and the study has been registered to ClinicalTrials.gov (Nbib3310502).

**Table 1:** Characteristics of the study participants. Data presented as mean ± s.d.

	Men	Women	All	*P* (men vs women)
Number of participants	216	110	326	
Age (years)	40.2 ± 15.3	53.4 ± 16.8	44.6 ± 17.0	<0.001
Body mass index (kg/m^2^)	26.8 ± 5.1	28.7 ± 7.4	27.4 ± 6.0	0.170
Fasting plasma glucose (mmol/L)	5.5 ± 0.5	5.6 ± 0.6	5.6 ± 0.5	0.907
Fasting serum insulin (μU/mL)	8.8 ± 6.2	8.9 ± 6.5	8.8 ± 6.3	0.815
Whole body GU (µmol/kg body weight/min)	28.7 ± 15.4	26.0 ± 13.0	27.8 ± 14.7	0.228
Femoral skeletal muscle GU (µmol/tissue kg/min)	42.9 ± 27.0	46.6 ± 29.7	44.1 ± 27.9	0.397
Liver GU (µmol/tissue kg/min)	21.7 ± 8.2	23.2 ± 10.2	22.4 ± 9.2	0.380
Subcutaneous adipose tissue GU (µmol/tissue kg/min)	10.9 ± 6.7	12.5 ± 7.0	11.6 ± 6.8	0.054
Intraperitoneal adipose tissue GU (µmol/tissue kg/min)	18.0 ± 10.2	24.9 ± 12.4	21.3 ± 11.8	<0.001
Endogenous glucose production (µmol/kg body weight/min)	7.0 ± 6.3	3.1 ± 4.6	5.1 ± 5.9	<0.001

GU, glucose uptake.

### Study design and methods

The PET studies were performed after an overnight fast. The subjects were instructed to avoid consumption of alcohol and caffeine for 12 h, and strenuous physical activity 24 h before the study. The subjects were in a supine position during the euglycemic–hyperinsulinemic clamp and PET scanning. A cannula was inserted in an antecubital vein for the infusion of glucose and insulin and the injection of [^18^F]FDG, and another cannula was inserted in the opposite upper extremity radial artery or antecubital vein that was warmed with a heating pillow to arterialize venous blood for blood sampling. Plasma glucose was maintained at euglycemia (~5 mmol/L) by a primed (first 4 min at 120 mU/body surface area m^2^/min and then 3 min at 80 mU/body surface area m^2^/min) and then continuous insulin infusion at 40 mU/body surface area m^2^/min and 20% glucose infusion based on plasma glucose measurements taken every 5–10 min (21). The rates of whole body GU (*M* value) were calculated from steady state and reported as the average of three 20-min intervals, starting after reaching euglycemia (median 60 min from the start of insulin infusion). [^18^F]FDG was injected at average 80 (interquartile range 60; 101) min from the start of insulin infusion, and dynamic scans were performed to get images of abdomen and femoral regions as previously described (25, 26). The timing when each region was scanned varied according the original PET research protocol. Plasma radioactivity was measured from arterial or arterialized blood samples.

#### Measurements of skeletal muscle, liver and adipose tissue glucose uptake

[^18^F]FDG was synthesized using a modified method of Hamacher *et al*. (27). PET-scanners ECAT 931/08 (Siemens Molecular Imaging, Inc., Knoxville, TN, USA), GE Advance, PET/CT Discovery VCT and PET/CT Discovery 690 (General Electric Medical Systems, Milwaukee, WI, USA) tomographs were used. The scanners were cross-calibrated against the same VDC-404 Dose calibrator (COMECER Netherlands, Joure, the Netherlands) to ensure the consistency of the results. All data obtained were corrected for dead time, decay and measured photon attenuation. The Bayesian iterative reconstruction algorithm, using median root prior with iterations and a Bayesian coefficient of 0.3, was used for image processing when possible (28). Tissue-specific GUs were measured by drawing the regions of interest (ROI) to quadriceps femoris, right lobe of the liver and abdominal subcutaneous and intraperitoneal adipose tissue. MRI or CT images were used as references for outlining the regions.

The rates of liver, skeletal muscle and adipose tissue GU were calculated by graphically analyzing plasma and tissue time–activity curves to quantify the fractional phosphorylation rate (*K*_i_) for [^18^F]FDG (29, 30). The rates of GU were calculated by multiplying *K*_i_ by the plasma glucose concentration and dividing by the tissue density and a lumped constant. A lumped constant corrects for the differences in transportation and phosphorylation of [^18^F]FDG and glucose. A lumped constant value of 1.0 for liver, 1.2 for skeletal muscle and 1.14 for adipose tissue were used (25, 26, 31).

#### Measurement of endogenous glucose production

EGP during the clamp study was measured using a previously validated method (32). In brief, ^18^F-FDG clearance adjusted with ^18^F-FDG lost to urine was used to determine glucose rate of disappearance. EGP was calculated by subtracting glucose infusion rate from glucose rate of disappearance. Possible changes in glucose level over the study were accounted by adding a space correction (21) to glucose infusion rate.

#### Biochemical analyses

Plasma glucose during the euglycemic–hyperinsulinemic clamp was determined in duplicate by the glucose oxidase method (Analox GM7 or GM9, Analox Instruments, London, UK). Serum insulin concentration, determined every 30 min during the clamp, was measured by a double antibody RIA (Phadeseph Insulin RIA kit, Pharmacia & Upjohn, Uppsala, Sweden) or automatized electro-chemiluminescence immunoassay (Cobas 8000, Roche Diagnostics GmbH), and serum FFA concentration, determined every 60 min during the clamp was measured using an enzymatic assay (ACS-ACOD, Wako Chemicals GmbH, Neuss, Germany).

#### Correlation testing and group comparisons

Statistical testing was done using IBM SPSS Statistics for Windows (version 23: IBM). Pearson correlation test was used to assess linear associations of the rates of tissue GU. GU values were log-transformed to achieve normal distribution. *T*-test or Mann–Whitney *U*-test was used to compare men and women or insulin-resistant and insulin-sensitive groups. The notched boxplots were created using ggplot2 package (33) of R statistical computing environment (version 3.4.1) (34).

#### Principal component analysis

Principal component analysis (PCA) and correlation testing were used to assess the association between the rates of tissue GU. PCA is a dimension reduction technique, which aims to combine several correlated variables to smaller number of components that explain most of the variation in the original variables thus allowing to find underlying patterns in the data (35). Eigenvalue 1.0 was used as a threshold value in PCA (36).

#### Receiver-operating characteristics curve analysis

We used receiver-operating characteristics (ROC) curve to find a cutoff value to divide our population to insulin-sensitive and -resistant subjects. Stern *et al.* (37) have previously provided a threshold for insulin resistance based on whole body GU per fat-free mass. Since we did not have body fat mass measurement required for this original threshold for all our subjects, we used ROC analysis to find a corresponding value expressed per body weight. The found cutoff 21 µmol/kg body weight/min had sensitivity 92% and specificity 99% with the original cutoff of 28 µmol/kg fat-free mass/min. It presented the lowest 10% of normal-weight subjects (BMI <25 kg/m^2^) and highest 16% of obese subjects (BMI ≥30 kg/m^2^) in our study cohort. Based on this cutoff, ROC curve analysis was used to find optimal thresholds for skeletal muscle and adipose tissue GU and EGP. Area under ROC curve over 0.7 is considered acceptable for discrimination (38). Analysis was performed separately according to sex where there was difference between sexes in tissue GUs or EGP.

## Results

### Associations between glucose uptake rates and EGP in different sexes

**Figure 1:**
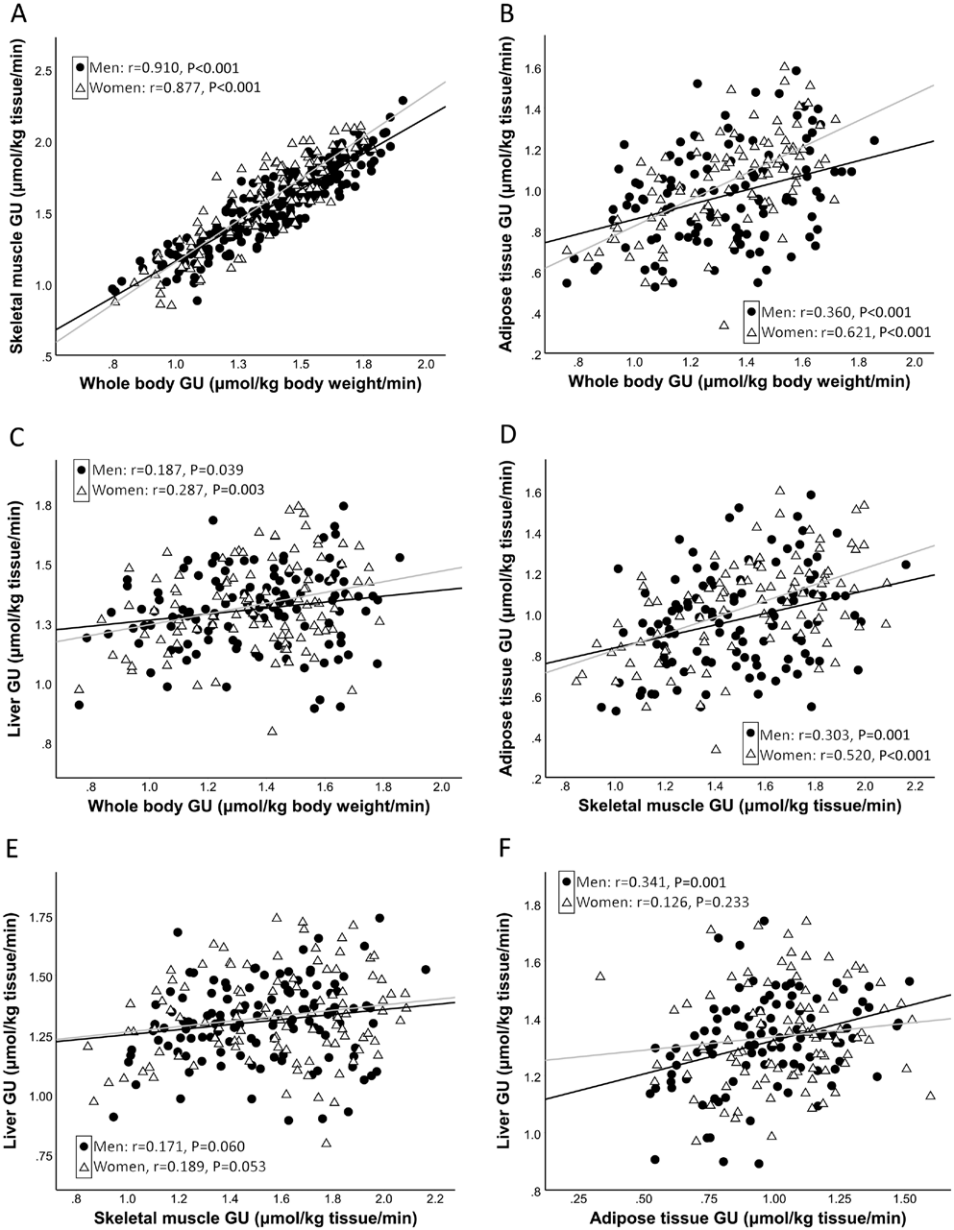
Correlation of whole body glucose uptake (GU) with skeletal muscle GU (A), subcutaneous adipose tissue GU (B) and liver GU (C); correlation of skeletal muscle GU with subcutaneous adipose tissue GU (D) and liver GU (E); and correlation of subcutaneous adipose tissue GU with liver GU (F). GU values are from log10 transformed variables. Black regression line: men; gray regression line: women.

In men and women, the *M* value correlated strongly and significantly with skeletal muscle GU (*r* = 0.910 and 0.877) and subcutaneous GU (*r* = 0.360 and 0.621, correspondingly; *P* = 0.015 between men and women) (Fig. 1), whereas the correlations of the *M* value with liver GU (*r* = 0.187 and 0.287) were considerably weaker. The correlation of skeletal muscle GU with subcutaneous GU was statistically significant in both genders (*r* = 0.303 in men and 0.520 in women), but correlation of skeletal muscle GU with liver GU was non-significant (*r* = 0.171 and 0.189, correspondingly) in both genders. BMI correlated inversely with GUs in all tissues examined (skeletal muscle *r* = −0.583, *P* < 0.001; liver *r* = −0.173, *P* = 0.009; subcutaneous adipose tissue *r* = −0.364, *P* < 0.001).

**Figure 2:**
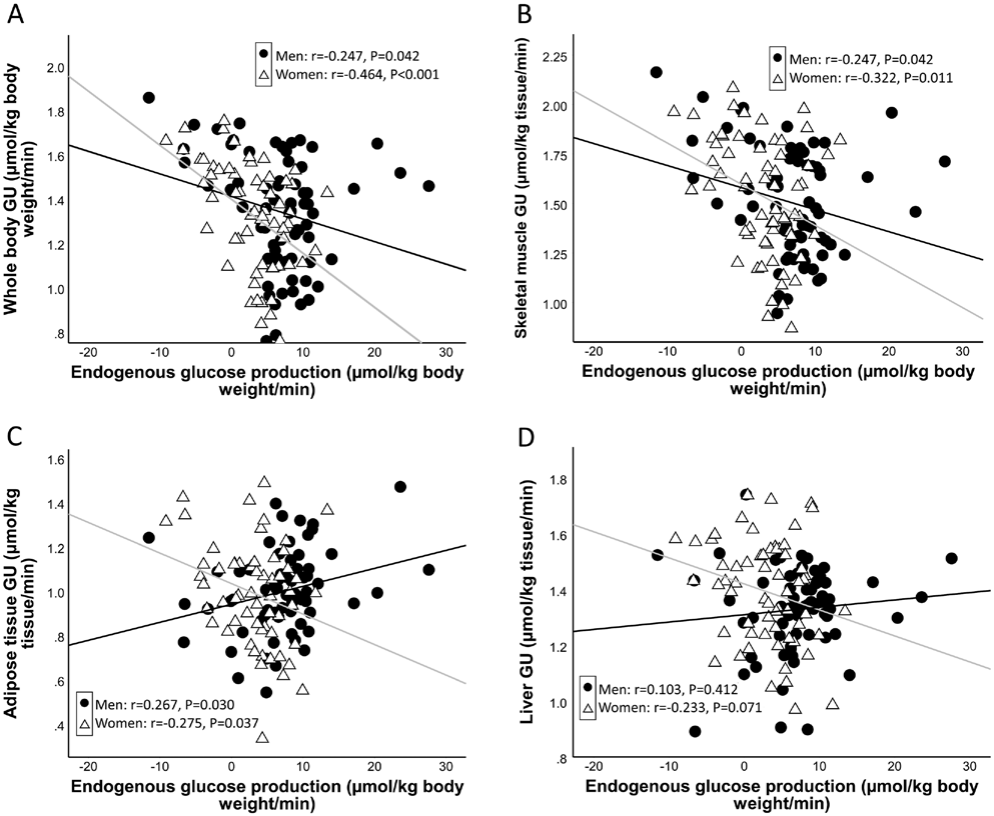
Correlation of endogenous glucose production with whole body glucose uptake (GU) (A), skeletal muscle GU (B), subcutaneous adipose tissue GU (C) and liver GU (D). GU values are from log10 transformed variables. Black regression line: men; gray regression line: women.

EGP during the clamp was inversely and significantly correlated with the *M* value in women but only weakly in men (*r* = −0.247 and −0.464 in men and women, respectively, Fig. 2). EGP correlated inversely with skeletal muscle GU in men and women (*r* = −0.247 and −0.322, correspondingly). Inverse correlation of EGP with subcutaneous adipose tissue and liver GU was observed in women (*r* = −0.275 and −0.233), but in men, there was a weak positive correlation of EGP with adipose tissue and liver GU (*r* = 0.267 and 0.103, respectively). When testing the correlation between EGP and intraperitoneal adipose tissue, there was no association in either men (*r* = 0.180, *P* = 0.147) or women (*r* = −0.090, *P* = 0.521).

A possible mediator between the associations of tissue GUs is the concentration of FFAs during the clamp. Significant inverse correlations of tissue GUs and FFA level during the clamp were observed in skeletal muscle (*r* = −0.333, *P* < 0.001), adipose tissue (*r* = −0.551, *P* < 0.001) and liver (*r* = −0.214, *P* = 0.005). FFAs during the clamp were higher in men than in women (0.12 (interquartile range 0.08–0.17) vs 0.05 (0.03–0.08) mmol/L, *P* < 0.001). Insulin and glucose levels during the clamp were 65 (57–82) mU/L and 5.1 (4.8–5.3) mmol/L.

### Cutoff values for muscle and adipose glucose uptake rates

**Figure 3:**
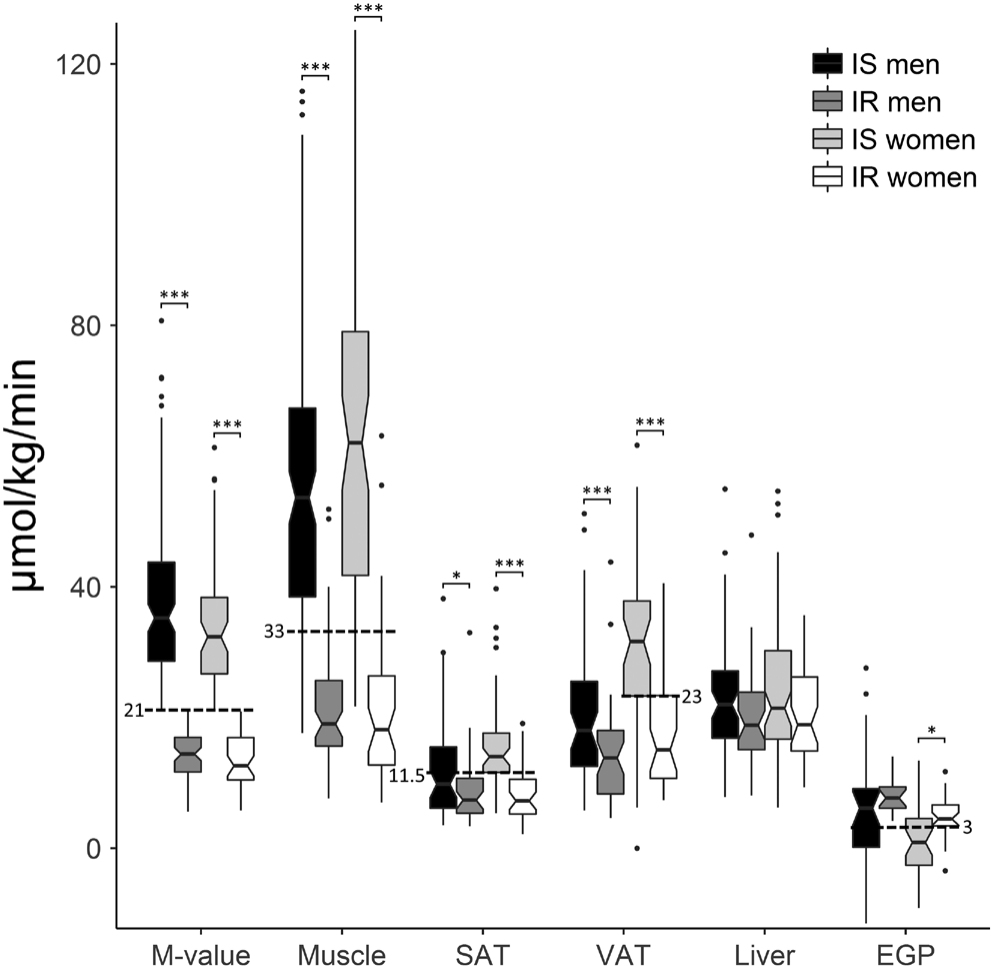
Whole body (*M* value), muscle, subcutaneous adipose tissue (SAT), intraperitoneal adipose tissue (VAT) and liver glucose uptake and endogenous glucose production (EGP) between insulin-sensitive (IS) and -resistant (IR) men and women. Dashed lines represent the optimal cutoff values between insulin-resistant and -sensitive individuals. The middle, bottom and top edges of the boxes represent median with 1st and 3rd quartiles, and notches are calculated as 1.58 × interquartile range/square root (*n*) (95% confidence interval for the median). If notches do not overlap, there is evidence for a difference between medians. The error bars extend to the furthest case inside 1.5 interquartile range from the box and outliers are presented as dots. ***P < 0.001, *P < 0.05.

Based on ROC curve analyses, the optimal cutoff points for tissue-specific insulin sensitivity were calculated for men and women. Skeletal muscle GU of 33 µmol/kg tissue/min separated insulin-sensitive and insulin-resistant subjects in ROC analysis (sensitivity 86% and specificity 89%) (Fig. 3). The cutoff for skeletal muscle GU presented the lowest 22% in normal-weight subjects and the highest 21% obese individuals. Skeletal muscle and adipose tissue were clearly insulin resistant in subjects with whole body insulin resistance, while insulin-stimulated liver GU was only slightly lower compared to the insulin-sensitive group (Fig. 3). We found that 11.5 µmol/kg tissue/min separated insulin-resistant subjects most accurately in both men and women, though area under ROC curve was less than 0.7 in men. Similarly, 3 µmol/kg body weight/min was found as optimal cutoff for EGP in both sexes but area under ROC curve was less than 0.7 in men. Liver GU between high and low insulin sensitivity groups was different only when not separating the population by sex and a good cutoff could not be found. Insulin suppression of EGP was worse in the insulin-resistant group, though the difference was not significant among men. Men had worse suppression of EGP compared to women (Table 1).

### Relationships between tissue insulin sensitivities, age, BMI and sex

Principal component analysis was used to study the relationships between tissue insulin sensitivities, age, BMI and sex. The results of PCA performed separately for men and women are shown in Table 2. The first principal component, PC1 (Model 1), including age, BMI and skeletal muscle and subcutaneous adipose tissue GU, explained 45% of the variation in men and 49% in women. Both skeletal muscle and adipose tissue GU had high positive and BMI negative loadings on PC1. PC2 was characterized by the effects of age in both genders. When combining men and women in PCA, the results remained largely unchanged. After adding liver GU in the model (Model 2), the variance explained was reduced to 38% in men and 39% in women attributable to the differences between the genders in liver GU (high loading only in men). The loading of liver GU on PC1 was substantially lower than those of skeletal muscle and adipose tissue in women suggesting a gender difference in the contribution of liver GU to the loadings on PC1. PC2 explained 27 and 32% of the variation in men and women, correspondingly, and was characterized by the effect of age on the rates of GU in adipose tissue and liver in men, but only on liver GU in women. In PCA analysis including both genders, the three PCs explained 72% of the variation. We performed similar PCA using intraperitoneal adipose tissue GU instead of abdominal subcutaneous adipose tissue GU and received largely similar results (Supplementary Table 1, see section on supplementary data given at the end of this article).

**Table 2:** Principal component analysis. Loadings in men and women indicate the contribution of each variable (GU, glucose uptake) to principal components (PC1, PC2, PC3).

GU (μmol/kg tissue/min)	Men		Women		All		
	PC1	PC2	PC1	PC2	PC1	PC2	PC3
Model 1							
Age (years)	−0.205	0.895	−0.006	0.952	0.095	0.693	–
Body mass index (kg/m^2^)	−0.857	0.023	−0.724	−0.489	−0.829	0.108	–
Gender	–	–	–	–	−0.033	0.822	–
Skeletal muscle GU	0.833	−0.091	0.850	0.126	0.830	0.015	–
Subcutaneous adipose tissue GU	0.585	0.617	0.841	−0.206	0.703	0.240	–
Variance explained (%)	45	30	49	30	38	25	–
Model 2							
Age (years)	−0.206	0.864	−0.039	0.881	−0.047	0.887	0.070
Body mass index (kg/m^2^)	−0.828	−0.057	−0.700	−0.442	−0.744	−0.278	0.292
Gender	–	–	–	–	0.061	0.076	0.959
Skeletal muscle GU	0.837	−0.051	0.844	0.162	0.848	0.039	0.047
Subcutaneous adipose tissue GU	0.458	0.662	0.846	−0.130	0.724	0.065	0.233
Liver GU	0.513	0.423	0.193	0.768	0.322	0.684	0.011
Variance explained (%)	38	27	39	32	32	22	18

We also performed PCA analysis where liver GU was replaced by EGP (Table 3). Skeletal muscle and adipose tissue GUs had positive loadings on PC1 in both genders, whereas EGP showed negative loading in women, and the lack of association in men (Table 3). In women, age and EGP loaded positively and BMI inversely on PC2. Men showed a similar pattern except for BMI, which had a weak association with EGP. Age had a positive loading with subcutaneous fat GU and EGP. When intraperitoneal adipose tissue GU was used in PCA, men had similar results when compared to the analysis using subcutaneous adipose tissue GU, but in women, intraperitoneal adipose tissue GU was not associated with EGP (Supplementary Table 2).

**Table 3:** Principal component analysis. Loadings in men and women indicate the contribution of each variable (GU, glucose uptake) to principal components (PC1, PC2, PC3). Liver GU was replaced by endogenous glucose production (EGP).

GU/EGP (μmol/kg tissue/min)	Men		Women		All		
	PC1	PC2	PC1	PC2	PC1	PC2	PC3
Model 1							
Age (years)	−0.206	0.741	0.079	0.909	0.241	−0.131	0.884
Body mass index (kg/m^2^)	−0.901	−0.117	−0.541	−0.774	−0.855	0.157	−0.232
Gender	–	–	–	–	−0.156	0.667	0.602
Skeletal muscle GU	0.816	−0.314	0.783	0.294	0.804	0.341	−0.127
Subcutaneous adipose tissue GU	0.562	0.644	0.765	0.147	0.703	−0.079	0.119
EGP	0.024	0.719	−0.684	0.547	−0.056	−0.886	0.159
Variance explained (%)	37	32	39	37	33	23	21

## Discussion

The associations of insulin sensitivity measured by the rates of GU into skeletal muscle, adipose tissue, and liver using the PET technique has not been previously investigated in a single study including a large number of participants. First, we showed that the rates of GU in skeletal muscle and adipose tissue were strongly correlated with each other and with the rates of whole body GU. By contrast, the correlation of liver GU with whole body GU was substantially weaker, and the correlation with skeletal muscle GU was not statistically significant. Secondly, EGP inversely correlated with whole body and skeletal muscle GU in both genders, and with subcutaneous adipose tissue and liver GU in women, but in men, EGP positively correlated with adipose tissue GU, but not with liver GU. Thirdly, PCA analysis (Table 2) demonstrated that the rates of GU in skeletal muscle, adipose tissue and liver had high loadings on PC1, and that obesity was negatively associated with the rates of GU similarly in all insulin-sensitive tissues in both men and women. Age was positively associated with GU in adipose tissue and in liver in men, but only with GU in liver in women (PC2). Furthermore, we provided information about distribution of tissue GUs and EGP during insulin stimulation among insulin-resistant and -sensitive subjects and cutoff values, which may be useful for determining what values can be considered normal.

Skeletal muscle takes the major part of GU during insulin stimulation in healthy individuals. We used quadriceps femoris to represent skeletal muscle in our study because its large size, very high correlation with whole body GU (Fig. 1) and important role in daily activities. Proportion of GU into adipose tissue and liver is substantially smaller than in skeletal muscle (1). Given the similarities of insulin signaling in skeletal muscle and adipose tissue, it was not surprising that there was a strong correlation of the rates of GU in these tissues (39). Insulin also suppresses lipolysis in adipose tissue by decreasing FFA concentration in the circulating blood. Impaired insulin’s antilipolytic effect in adipose tissue leads to a high flux of FFAs into the liver, and consequently to an increase in triglyceride synthesis. Additionally, high levels of FFAs impair GU in skeletal muscle and adipose tissue (40, 41). In line with previous studies, FFA concentration during the euglycemic clamp in our study negatively correlated with the rates of GU in skeletal muscle, adipose tissue and liver.

In the postprandial state about one-third of ingested glucose is taken up by the liver and stored as glycogen in individuals with normal glucose tolerance (12). Elevated insulin levels stimulate insulin signaling in the liver that promotes glycogen synthesis and lipogenesis and suppresses glycogen breakdown (42). Hepatic gluconeogenesis from non-glucose precursors (lactate, pyruvate, glycerol and amino acids) and glycogen breakdown (glycogenolysis) are the two processes responsible for EGP (43). We found that there was no difference between men and women in liver GU, but men had higher EGP than women, which agrees with a previous study (19). Moreover, men had worse insulin-mediated suppression of lipolysis, which may contribute to the higher EGP (42). EGP correlated inversely and significantly with whole body GU and skeletal muscle GU in women, but only nominally in men.

By applying PC analysis, we wanted to determine if GU in major insulin-sensitive tissues is similarly changed during the euglycemic clamp. Skeletal muscle and adipose tissue GU had high loadings on PC1 but adding liver GU in Model 2 reduced total variation explained by this component (from 45 to 38% in men, from 49 to 39% in women). This suggests that the regulation of GU in the liver differs from that of skeletal muscle and adipose tissue GU without any major differences between men and women. When liver GU was replaced by EGP (another measure of liver insulin resistance) in PC analysis, EGP loaded weakly on PC1, whereas in women, EGP had a negative loading. In women, the effect of age and BMI on EGP was more pronounced than in men.

ROC analysis allowed us to calculate cutoff values which can be used to assess whether a person is insulin sensitive or resistant according to skeletal muscle (33 µmol/kg tissue/min) or subcutaneous adipose tissue GU (11.5 µmol/kg tissue/min) or EGP (3 µmol/kg body weight/min) during euglycemic–hyperinsulinemic clamp using insulin infusion rate of 40 mU/body surface area m^2^/min. Separation of the insulin-sensitive and -resistant groups was clear with the cutoff for skeletal muscle GU in the whole group and subcutaneous adipose tissue and EGP in women. Even though area under ROC curve was lower than 0.7 when calculating thresholds for subcutaneous adipose tissue and EGP measurements in men, the found cutoffs were similar as for women. Thus, these cutoffs may be useful when interpreting quantitative values of tissue GU or EGP in future studies.

This study has some limitations. After a meal, glucose gradient between portal vein and liver cells has major impact on liver glucose uptake; however, we could not measure this effect due to lack of reliable model for ^18^F-FDG and glucose kinetics in the non-steady glycemic state after meal. Nevertheless, by using euglycemic hyperinsulinemia, we were able to study insulin sensitivity of liver GU, which contributes to glucose clearance after a meal. In addition, it would have been more elegant to compare GU and EGP between fasting state and hyperinsulinemia to measure insulin sensitivity. However, because this study was conducted by combining previously performed research, where several substudies were interventions, radiation exposure and time and resource constraints limited the possibility to include fasting GU measurement to the protocols. Still, since insulin-stimulated GU can be several times higher compared to GU at fasting state in skeletal muscle and adipose tissue and 2-fold higher in the liver, and EGP may be completely suppressed, major part of the variation in these measures in this study can be explained by insulin sensitivity.

In conclusion, we have provided threshold values, which can be used to identify tissue-specific insulin resistance. Our results suggest that insulin resistance measured by GU is partially similar in all insulin-sensitive tissues, skeletal muscle, adipose tissue and liver and are affected by obesity, aging and gender. Liver GU and EGP did not explain the variance beyond that contributed by skeletal muscle and adipose tissue GU, suggesting that the mechanisms responsible for liver insulin resistance differ from those present in skeletal muscle and adipose tissue. A crosstalk between insulin-sensitive tissues, especially insulin’s antilipolytic effects in adipose tissue affecting skeletal muscle GU, and liver function and EGP, are at least in part explaining the differences between the insulin-sensitive tissues.

## Supplementary Material

Supporting Table 1

Supporting Table 2

## Declaration of interest

The authors declare that there is no conflict of interest that could be perceived as prejudicing the impartiality of this study.

## Funding

The study has been conducted at the Center of Excellence in Cardiovascular and Metabolic Diseases, supported by the Academy of Finland, the University of Turku, Turku University Hospital and Åbo Akademi University. It was supported also by the Finnish Cultural Foundation, Varsinais-Suomi Regional fund, Yrjö Jahnsson Foundation, Turku University Foundation, University of Turku, Combined Research Fund and Oskar Öflund Stiftelse.

## Author contribution statement

M J H contributed to conception of the work, analysis of the data, interpretation of the results and writing the manuscript. A L R, M B, K A V, J C H and K K K acquired data and revised the manuscript. P N contributed to conception of the work, interpretation of the results, revised the manuscript and supervised the work. All authors accepted the manuscript to be published.
