# Supplementary material for: Insulin-stimulated glucose uptake in skeletal muscle, adipose tissue and liver: a positron emission tomography study
Source: Eur J Endocrinol. 2018 Mar 7;178(5):523–31. doi: 10.1530/EJE-17-0882 (PMC5920018; doi:10.1530/EJE-17-0882)
Supplement: Supporting Table 1 [file eje-178-523-t001.pdf]

Supplementary table 1. Principal component analysis. Loadings in men and women indicate the contribution of each variable (GU=glucose uptake) to principal components (PC1, PC2).

**Model 1**	Men		Women		All	
GU(μmol/kg tissue/min)	PC1	PC2	PC1	PC2	PC1	PC2
Age (years)	-0.316	-	0.568	-	0.093	0.677
Body mass index (kg/m^2)	-0.857	-	-0.890	-	-0.874	0.079
Gender	-	-	-	-	-0.091	0.850
Skeletal muscle GU	0.779	-	0.751	-	0.814	-0.004
Intraperitoneal adipose tissue GU	0.661	-	0.795	-	0.652	0.472
Variance explained	47%	-	58%	-	37%	28%
**Model 2**						
Age (years)	-0.070	0.919	0.151	0.864	0.271	0.574
Body mass index (kg/m^2)	-0.847	0.021	-0.770	-0.398	-0.881	0.024
Gender	-	-	-	-	-0.195	0.857
Skeletal muscle GU	0.806	-0.183	0.868	0.002	0.768	0.059
Intraperitoneal adipose tissue GU	0.526	0.419	0.800	0.208	0.519	0.561

Liver GU	0.535	0.281	0.154	0.799	0.497	0.307
Variance explained	39%	23%	41%	32%	33%	25%
