# Supplementary material for: Insulin-stimulated glucose uptake in skeletal muscle, adipose tissue and liver: a positron emission tomography study
Source: Eur J Endocrinol. 2018 Mar 7;178(5):523–31. doi: 10.1530/EJE-17-0882 (PMC5920018; doi:10.1530/EJE-17-0882)
Supplement: Supporting Table 2 [file eje-178-523-t002.pdf]

Supplementary table 2. Principal component analysis. Loadings in men and women indicate the contribution of each variable (GU=glucose uptake) to principal components (PC1, PC2, PC3). Liver GU was replaced by endogenous glucose production (EGP).

**Model 1**	Men		Women		All		
GU/EGP (μmol/kg tissue/min)	PC1	PC2	PC1	PC2	PC1	PC2	PC3
Age (years)	-0.226	0.796	0.848	0.280	0.868	0.121	-0.133
Body mass index (kg/m2)	-0.890	-0.155	-0.930	-0.172	-0.352	-0.803	0.291
Gender	-	-	-	-	0.619	-0.274	0.575
Skeletal muscle GU	0.854	-0.228	0.739	-0.352	0.038	0.893	0.235
Intraperitoneal adipose tissue GU	0.375	0.758	0.856	-0.131	0.799	0.340	0.048
EGP	-0.048	0.605	0.031	0.954	0.089	-0.105	-0.909
Variance explained	34%	33%	57%	23%	31%	28%	22%
